# Prone positioning does not improve outcomes of intubated patients with pneumocystis pneumonia and moderate–severe acute respiratory distress syndrome: a single-center, retrospective, observational, cohort study

**DOI:** 10.1186/s40001-024-01868-7

**Published:** 2024-05-03

**Authors:** Zhen Wang, Yuyan Zhou, Min Zhu, Faping Wang, Yubei Zhou, He Yu, Fengming Luo

**Affiliations:** 1 Department of Respiratory Care, Sichuan University West China Hospital, Chengdu, Sichuan China; 2 State Key Laboratory of Respiratory Health and Multimorbidity, West China hospital, Sichuan University, Chengdu, China; 3 Department of Pulmonary and Critical Care Medicine, West China Hospital, Sichuan University, Chengdu, China 610041; 4 Laboratory of Pulmonary Immunology and Inflammation, Department of Respiratory and Critical Care Medicine, Frontiers Science Center for Disease-Related Molecular Network, West China Hospital, Sichuan University, Chengdu, 610041 Sichuan People’s Republic of China

**Keywords:** PJP, Prone positioning, Moderate–severe ARDS

## Abstract

**Background:**

Pneumocystis pneumonia is an uncommon precipitant of acute respiratory distress syndrome and is associated with high mortality. Prone positioning ventilation has been proven to reduce mortality in patients with moderate–severe acute respiratory distress syndrome. We investigated the effect of prone positioning on oxygenation and mortality in intubated patients with pneumocystis pneumonia comorbid with moderate–severe acute respiratory distress syndrome.

**Methods:**

In this single-center, retrospective, observational, cohort study, eligible patients were enrolled at West China Hospital of Sichuan University from January 1, 2017, to December 31, 2021. Data on demographics, clinical features, ventilation parameters, arterial blood gas, and outcomes were collected. Patients were assigned to the prone cohort or supine cohort according to whether they received prone positioning ventilation. The main outcome was 28-day mortality.

**Findings:**

A total of 79 patients were included in the study. Sixty-three patients were enrolled in the prone cohort, and 16 patients were enrolled in the supine cohort. The 28-day mortality was 61.9% in the prone cohort and 68.8% in the supine cohort (*P* = 0.26), and 90-day mortality was 66.7% in the prone cohort and 68.8% in the supine cohort (*P* = 0.55). Patients in the supine cohort had fewer invasive mechanical ventilation days and more ventilator-free days. The incidence of complications was higher in the prone cohort than in the supine cohort.

**Conclusions:**

In patients with pneumocystis pneumonia and moderate–severe acute respiratory distress syndrome, prone positioning did not decrease 28-day or 90-day mortality.

*Trial registration* ClinicalTrials.gov number, ChiCTR2200063889. Registered on 20 September 2022, https://www.chictr.org.cn/showproj.html?proj=174886.

**Supplementary Information:**

The online version contains supplementary material available at 10.1186/s40001-024-01868-7.

## Introduction

Pneumocystis pneumonia (PCP) is an opportunistic pulmonary infection that occurs in immunocompromised or HIV-infected individuals or patients with malignancy [1, 2]. Patients with PCP may develop symptoms, such as fever, cough, progressive dyspnea, and hypoxemia [3, 4]. PCP is an uncommon precipitant of acute respiratory distress syndrome (ARDS) [5], but is associated with a high mortality rate of up to 0% to 20% in HIV-infected patients and 30–75% in non-HIV-infected patients [1, 4–6]. However, quality evidence regarding how to ventilate patients with critical PCP is lacking.

The fungus *Pneumocystis jirovecii* is transmitted through the airborne route [4], and computed tomography (CT) scans of PCP show predominant ground-glass opacification (GGO) with diffuse, bilateral and central locations in the early stage and patchy or focal consolidation in the middle or late stages [7–10], indicating a more homogeneous ventilation distribution between the ventral lung, middle lung and dorsal lung than “typical” ARDS. Prone positioning ventilation (PPV) has been used to treat hypoxemia in patients with severe or moderate ARDS for several years [11]. The principle underlying PPV for remedying hypoxemia involves improving ventilation and alveolar recruitment in the dorsal lung, the so-called “dependent pulmonary units”, thus improving the ventilation/perfusion ratio and reduction in intrapulmonary shunting [12–14]. However, it is unclear whether a patient with PCP can benefit from PPV treatment. To our knowledge, there is no study or case report available referring to the efficacy of prone positioning in PCP patients.

In this retrospective, cohort study, it was hypothesized that PPV would not improve oxygenation or reduce mortality in intubated patients with PCP and moderate–severe ARDS.

## Material and methods

### Study design and participants

In this single-center, retrospective, observational, cohort study, we recruited eligible patients admitted to the medical intensive care unit (MICU) and center intensive care unit (CICU) of West China Hospital of Sichuan University, a large tertiary teaching hospital, between January 1, 2017, and December 31, 2021.

Patients were eligible to be included in the study if they were between 18–85 years of age, were diagnosed with PCP by etiological examination (fungal smear/cultures or metagenomics next generation sequencing) of sputum or bronchoalveolar lavage fluid, and received invasive mechanical ventilation targeted at moderate–severe ARDS for less than 72 h prior to admission to the ICU.

Exclusion criteria were pregnancy, advanced carcinoma or massive hemorrhage, and invasive mechanical ventilation less than 72 h after admission to the ICU.

Patients who received PPV treatment were enrolled in the prone cohort, and those who received no PPV were enrolled in the supine cohort.

### PPV and mechanical ventilation strategies

The PPV procedure conducted in our study was based on the protocol published as part of the PROSEVA trial [15]. The durations mostly occurred for 12 h [11, 16] and were extended to 16–18 h when necessary [15, 17]. Patients were eligible to receive PPV if the PaO_2_/FiO_2_ ratio was lower than 200 mmHg (typically lower than 150 mmHg), upon evaluation by their attending physician. Complications leading to immediate termination of PPV included unexpected artificial airway extubation or obstruction, hemoptysis, oxygen saturation less than 85% on pulse oximetry for more than 2 min when the FiO_2_ was 1.0, cardiac arrest, a heart rate of less than 50 beats or more than 160 beats per minute for more than 30 s, a systolic blood pressure of less than 90 mm Hg for more than 5 min, or any other life-threatening incidents.

Mechanical ventilation was delivered according to the ALVEOLI study [18], with end-inspiratory plateau pressure (Pplat) maintained below 30 cm H_2_O, and volumes guaranteed between 6–8 mL/kg PBW or 4–6 mL/kg PBW during extracorporeal membrane oxygenation (ECMO) therapy. Permissive hypercapnia was flexibly performed when lung-protective ventilation was impeded, with pH values of 7.30–7.35 and 7.25–7.35 in patients with chronic obstructive pulmonary disease.

### Outcome measures

The primary end point was mortality at 28 days. The secondary endpoints were mortality at 90 days, the rate of successful extubation on day 28, the length of invasive mechanical ventilation days and ventilator-free days at 28 days, the length of ICU stay on day 90, the pneumothorax incidence rate, and the tracheotomy rate at 90 days.

Successful extubation was defined as no reintubation for 48 h after extubation. In patients who had undergone tracheotomy, successful weaning from the ventilator was defined as the ability to breathe through the tracheostomy cannula for at least 48 h with oxygen therapy or high-flow oxygen therapy.

### Data source

We collected data via the hospital electronic health record system, including age, sex, body mass index, Sequential Organ Failure Assessment (SOFA) score, Simplified Acute Physiology Score II (SAPS II), smoking history, patient origin, coexisting conditions, coexisting germs in the respiratory system, anti-PCP drug before ICU admission, cointerventions, ventilator settings and arterial blood gas (ABG) parameters, laboratory tests and radiological characteristics.

Chronic obstructive pulmonary disease, asthma, and interstitial lung disease were recorded as chronic bronchopulmonary disease, while patients using immuno-suppressive drugs, such as cyclosporin A, protopic, mycophenolate mofetil, azathioprine, and prednisone were considered receiving immuno-suppressive therapies.

For the prone cohort, ventilator settings, ABG values and adverse events were collected for every single PPV session at three time points: 0–2 h before prone positioning (SPV1), 0–2 h after shifting to prone positioning (PPV) and 1–2 h after supervised repositioning (SPV2).

### Statistical analysis

Efficacy analyses were performed using the full analysis set, and patients who received at least one PPV session were assigned to the prone cohort. To investigate the effect of PPV on oxygenation, we compared patient PaO_2_/FiO_2_ ratios between the SPV1 and PPV time points in the first PPV session and compared the PaO_2_/FiO_2_ ratio between cohorts during the first 3 ICU days. We compared the PaO_2_/FiO_2_ of the PPV versus SPV1 time points. The effects of prone positioning were classified into three degrees according to the change in the PaO_2_/FiO_2_ ratio between PPV and SPV1 as follows: type A represented conditions for which the PaO_2_/FiO_2_ ratio increased by over 15%, type B represented conditions for which the PaO_2_/FiO_2_ ratio changed between − 15% and + 15%, and type C represented conditions for which the PaO_2_/FiO_2_ ratio decreased by over 15%. Adverse events occurring in the first 3 ICU days in both cohorts were compared. We chose patients who received at least 3 consecutive PPV sessions in the prone cohort and all patients from the supine cohort to compare PaO_2_/FiO_2_ trends between cohorts, and PaO_2_/FiO_2_ values were gathered corresponding to the supine position after PPV. The analysis was performed with repeated-measures ANOVA and the ΔPaO_2_/FiO_2_ from D2 to D1 and D3 to D1 were compared by Student’s t test.

Continuous variables are expressed as means with standard deviations (SD) or quartiles (upper quartile Q1, median Q2, lower quartile Q3), according to the distribution, and categorical variables are described as percentages. Continuous variables were compared between groups with Student’s t test or Mann–Whitney *U* tests, based on the distribution, and categorical variables were compared by Chi-square test or Fisher’s exact test. Patient survival was analyzed by the Kaplan–Meier method and compared between groups with the use of the log-rank test. Factors associated with mortality were identified with Cox proportional hazards regression, and the results were expressed as hazard ratios (HRs) with 95% CI. The proportional hazard assumption was verified using the Schoenfeld test.

Missing data were appended by mean completer or regression completer according to their randomization. For patients who were transferred to other hospitals, we contacted their family members by phone to obtain information on their outcomes.

The statistical analysis was performed using R software (R for Mac, version 4.2.3) and Prism (for Mac, version 9.4.1). All reported *P* values were two-sided, and a *P* value of less than 0.05 was considered to indicate statistical significance.

## Results

### Patient characteristics

**Fig. 1:**
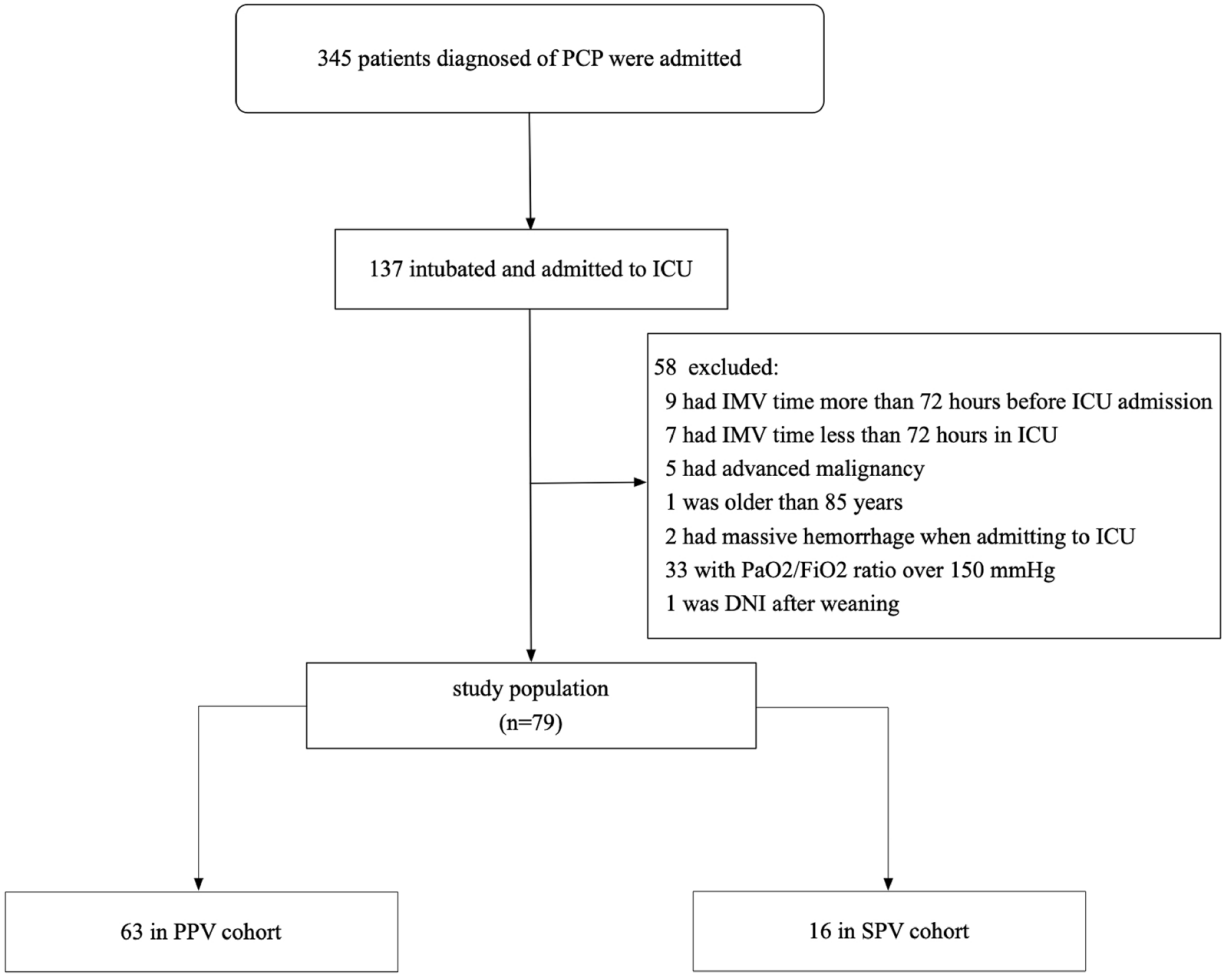
Study profile. *PCP* pneumocystis pneumonia, *ICU* intensive care unit, *DNI* do not intubate, *PPV* prone positioning ventilation, *SPV* supine positioning cohort

From January 1, 2017, to December 31, 2021, a total of 137 intubated patients with PCP were selected, 58 patients were subsequently excluded, and 79 patients were included in the current study (Fig. 1). Patients in the prone cohort received at least one prone positioning session (median 5, IQR 2–7) and underwent continuous PPV with an average duration of 12.6 ± 2.8 h. Table 1 shows the characteristics, cointerventions, ventilator settings and arterial blood gas measurements of the study population. There was no significant difference between the two cohorts. Laboratory tests and typical radiologic characteristics of the study population are provided in Table 2. There were no significant differences between the two cohorts.

**Table 1:** Characteristics of the participants at inclusion in the study

Characteristic	Supine cohort (*n* = 16)	Prone cohort (*n* = 63)	*P* value
Age, years	52.9 (13.5)	53.6 (14.5)	0.861
Male sex, no. (%)	10 (62.5)	36 (57.1)	0.917
Body mass index (kg.m^−2^), Q2 (Q1, Q3)	21.3 (20.5, 21.3)	21.9 (19.8, 23.4)	0.152
SOFA, Q2 (Q1, Q3)	8.3 (6.0, 12.0)	8.0 (5.0, 9.0)	0.619
SAPS II	39.4 (10.2)	37.3(8.1)	0.439
Smoking history, no. (%)			
Never smoked	9 (56.3)	43 (68.3)	0.504
Ever smoker	3 (18.8)	11 (17.5)	
Active smoker	4 (25.0)	9 (14.3)	
Patient origin, no. (%)			
Emergency room	6 (37.5)	28 (44.4)	0.282
Inpatient department	9 (56.3)	35 (55.6)	
Other	1 (6.3)	0	
Coexisting conditions, no. (%)			
Diabetes	4 (25.0)	16 (25.4)	1.000
Chronic bronchopulmonary disease	4 (25.0)	10 (15.9)	0.466
Chronic heart disease	0	3 (4.8)	1.000
Chronic renal disease	8 (50.0)	22(34.9)	0.387
Solid organ transplant	4 (25.0)	9 (14.3)	0.449
Malignancy	1 (6.3)	9 (14.3)	0.677
Hypogammaglobulinemia	7 (43.8)	22 (34.9)	0.568
Immuno-suppressive therapies for at least 3 months	10 (62.5)	33 (52.4)	0.578
HIV positive	1 (6.3)	2 (3.2)	0.498
Coexisting germ, no. (%)			
Bacteria	11 (68.8)	37 (58.7)	0.572
Other fungal	6 (37.5)	17 (37.0)	0.535
Virus	12 (75.0)	41 (65.1)	0.559
Mycoplasma/chlamydia	1 (6.3)	1 (1.6)	0.366
Anti-PCP drug before ICU admission, no. (%)	10 (62.5)	50 (79.4)	0.194
NIV before intubation, no(%)	11 (68.8)	54 (85.7)	0.144
Co-interventions, *n* (%)			
Vasopressors	9 (56.3)	25 (39.7)	0.268
Neuromuscular blockers	8 (50.0)	36 (57.1)	0.779
Renal replacement therapy	4 (25.0)	21 (33.3)	0.764
ECMO therapy	0	6 (9.5)	0.338
Ventilator settings and arterial blood gas measurements at the time of inclusion^a^			
Vt, ml	411 (35.0)	400 (48.0)	0.128
Vt/per kg of PBW	6.9 (0.4)	6.7 (0.9)	0.749
PEEP, cmH_2_O, Q2 (Q1, Q3)	11.0 (10.0, 12.5)	10.0 (8.5,12.0)	0.382
FiO_2_	0.8 (0.2)	0.8 (0.2)	0.754
PaO_2_, mmHg, Q2 (Q1, Q3)	71.5 (61.7, 83.5)	75.3 (65.4, 102.0)	0.157
PaCO_2_, mmHg	45.3 (15.0)	47.8 (17.4)	0.566
pH, Q2 (Q1, Q3)	7.31 (7.29,7.38)	7.37 (7.30, 7.42)	0.247
PaO_2_/FiO_2_, mmHg, Q2 (Q1, Q3)	97.1 (72.7, 115.2)	110.3(94.1, 130.4)	0.075
Lactate, mmol/L	1.9 (0.82)	1.9 (0.94)	0.930
Plasma bicarbonate, mmol/L, Q2 (Q1, Q3)	19.9 (19.2, 24.7)	23.1(20.2, 27.7)	0.182

Data are mean (SD), *n* (%), or median (IQR). Student’s *t* test or Mann–Whitney rank-sum test and Chi-square or Fisher exact test, were used as appropriate

*SOFA* Sequential Organ Failure Assessment, *SAPS II* Simplified Acute Physiology Score II, *PCP* Pneumocystis pneumonia, *NIV* non-invasive ventilation, *ECMO* extracorporeal membrane oxygenation, *PBW* predicted body weight, *FiO*_*2*_ the fraction of inspired oxygen, *PaO*_*2*_ partial pressure of arterial oxygen, *PaCO*_*2*_ partial pressure of arterial carbon dioxide, *PEEP* positive end-expiratory pressure

^a^We excluded 6 patients in prone positioning cohort who received ECMO therapy

**Table 2:** Laboratory tests and radiologic characteristics of patients in the study

	Supine cohort (*n* = 16)	Prone cohort (*n* = 63)	*P* value
Laboratory test			
CD4 count	83.4 (42.6)	94.5 (58.1)	0.395
Albumin, Q2 (Q1, Q3)	28.1 (23.8, 32.5)	27.6 (27.6, 27.6)	0.855
LDH	757.8 (498.1)	788.1 (475.0)	0.828
WBC, Q2 (Q1, Q3)	8.5 (6.5, 13.3)	8.2 (4.2, 12.3)	0.774
Procalcitonin	17.1 (25.9)	5.3 (9.6)	0.093
CRP, Q2 (Q1, Q3)	133.8 (114.8)	166.8 (106.5)	0.309
Radiologic characteristics, *n* (%)			
Ground glass opacity	9 (56.3)	27 (42.9)	0.405
Lung nodules	5 (31.2)	12 (19.0)	0.316
Interstitial markings	8 (50.0)	39 (61.9)	0.561
Consolidation	9 (56.3)	30 (47.6)	0.736
Focal	1	4	
Non-focal	8	26	

*LDH* lactate dehydrogenase, *WBC* white blood cell, *CRP* C-reactive protein

### Outcomes

**Fig. 2:**
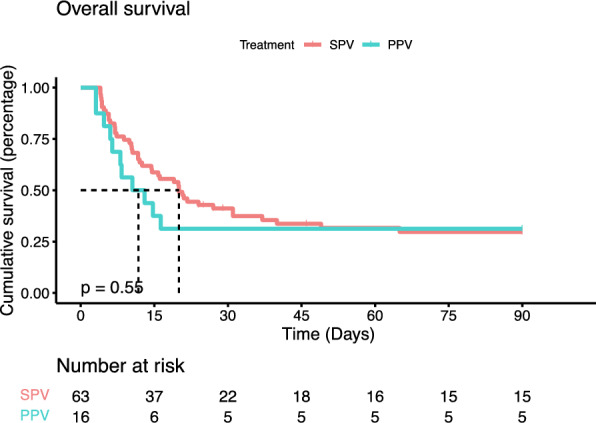
Kaplan–Meier plot of the probability of survival at day 90. *PPV* prone positioning ventilation cohort, *SPV* supine positioning ventilation cohort

Table 3 shows the outcomes of the patients in the two cohorts. There was no significant difference between the supine cohort and prone cohort in mortality on day 28 (68.8% vs 61.9%, *P* = 0.26) or day 90 (68.8% vs 66.7%, *P* = 0.55). Figure 2 shows the Kaplan–Meier plot of both groups on day 90.

**Table 3:** Primary and secondary outcomes of patients in the study

Outcome	Supine cohort (*n* = 16)	Prone cohort (*n* = 63)	*P* value
Mortality at d 28	11 (68.8)	39 (61.9)	0.260
Mortality at d 90	11 (68.8)	42 (66.7)	0.550
Successful extubation at day 28	5 (31.3)	11 (17.5)	0.295
Length of IMV days at day 28, d	8.6 (3.7)	16.0 (8.7)	0.002
Ventilator-free days at day28	18.0 (16, 18.5)	0 (0, 10.5)	0.001
Length of ICU stay, assessed at day 90-days, Q2 (Q1, Q3)			
Survivors	12.5 (9.0, 12.0)	8.1 (5.4, 11.8)	0.173
Non-survivors	19.0 (11.6, 39.0)	11.7 (6.5, 20.5)	0.057
Pneumothorax	0	11 (17.5%)	0.108
Tracheotomy of survivor at day 90	0	11 (17.5%)	0.108

*IMV* invasive mechanical ventilation

Patients in the supine cohort had fewer invasive mechanical ventilation days and more ventilator-free days than those in the prone cohort at 28 days. The rate of successful extubation, ICU stay days, incidence of pneumothorax and rate of tracheotomy did not differ significantly between the two groups.

Six patients received ECMO (all in the prone cohort) with 100% mortality on day 90.

### Prone positioning ventilation

**Fig. 3:**
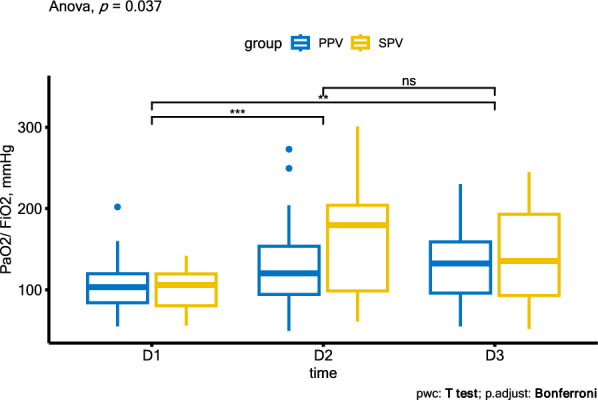
PaO_2_/FiO_2_ ratio of patients in both cohort in the first 3 days. *PPV* prone positioning ventilation, *SPV* supine positioning ventilation. *P < 0.05 versus D1; NS, no significant difference

The PaO_2_/FiO_2_ ratio of patients in both cohorts was higher at day 2 and day 3 than at day 1 (Fig. 3), but when comparing ΔPaO_2_/FiO_2_, there was no significant difference between the two cohorts on day 2 or day 3 (Table 4).

**Table 4:** Changes of PaO_2_/FiO_2_ ratio of patients in both cohorts in the first 3 days

Time	Prone cohort (*n* = 38)		Supine cohort (*n* = 16)		*P* value of change between cohorts
	PaO_2_/FiO_2_, mmHg	Change from Day 1 (95% CI)	PaO_2_/FiO_2_, mmHg	Change from Day 1(95% CI)	
Day 1	102.9 (31.9)	…	101.3 (26.3)	…	…
Day 2	127.8 (49.7)	24.9 (7.7, 42.0)	166.9 (78.0)^*^	65.6 (27.1, 104.0)	0.053
Day 3	136.7 (66.2)	33.8 (10.8, 56.7)	140.2 (58.4)	38.9 (10.9, 66.8)	0.770

For patients in the prone cohort, we only included those who received 3 consecutive sessions of PPV

^*^P < 0.05 versus the prone cohort

**Fig. 4:**
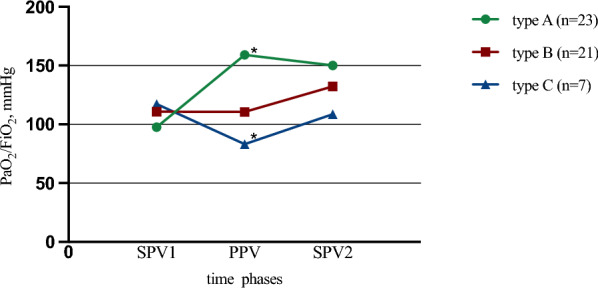
PaO_2_/FiO_2_ trajectory in the first PPV sessions. SPV1, 0–2 h before prone positioning ventilation; PPV, 0–2 h after shifting to prone positioning ventilation; SPV2, 1–2 h after resupervised positioning. Type A, PaO2/FiO2 ratio increased by over 15%; type B, PaO2/FiO2 ratio changed between − 15% and + 15%; type C, PaO2/FiO2 ratio decreased by over 15%. * P < 0.05 versus SPV1

For the first PPV, sessions were terminated earlier for 6 patients (1 for arrhythmia, 3 for low SpO_2_ and 2 for shock), and 6 were excluded due to receiving ECMO treatment. Of the 51 eligible patient sessions, the PaO_2_/FiO_2_ ratio was 105.3 mmHg (SD 28.5) in the SPV1 phase and 115.5 mmHg (IQR 91.5–147.7) in the PPV phase, and there was no significant difference between the SPV1 phase and PPV phase (*P* = 0.1173). The per-time trajectory of PaO_2_/FiO_2_ at the three study time phases is shown in Fig. 4.

The complications of the first 3 PPV sessions are listed in Table 5. In the first PPV session, SpO_2_ decreased by ≥ 4% in 29 (46%) patients, which was significantly higher than that of patients in the SPV cohort on day 1 (*P* = 0.0033).

**Table 5:** Adverse events of both cohort in the first 3 sessions of prone positioning or first 3 days

	S1/D1		S2/D2		S3/D3	
	PPV cohort (*n* = 63)	SPV cohort (*n* = 16)	PPV cohort (*n* = 49)	SPV cohort (*n* = 16)	PPV cohort (*n* = 44)	SPV cohort (*n* = 16)
Arrhythmia, *n* (%)	0	1 (6.3)	3 (6.1)	1 (6.3)	2 (4.5)	1 (6.3)
Shock, *n* (%)	6 (9.5)	2 (12.5)	2 (4.1)	1 (6.3)	2 (4.5)	0
SpO2 < 85% for more than 2 min, *n* (%)	8 (12.7)	2 (12.5)	4 (8.2)	2 (12.5)	2 (4.5)	1 (6.3)
SpO2 decrease by ≥ 4%, *n* (%)	29 (46.0)^*^	1 (6.3)	13 (26.5)	2 (12.5)	4 (9.1)	0
Airway obstruction, *n* (%)	0	0	0	0	0	0
Unexpected extubation, *n* (%)	0	0	0	0	0	0
Early termination, *n* (%)	6 (9.5)	…	2 (4.1)	…	1 (2.3)	…

^*^P < 0.05 versus the supine cohort

### Factors associated with 28-day mortality

**Fig. 5:**
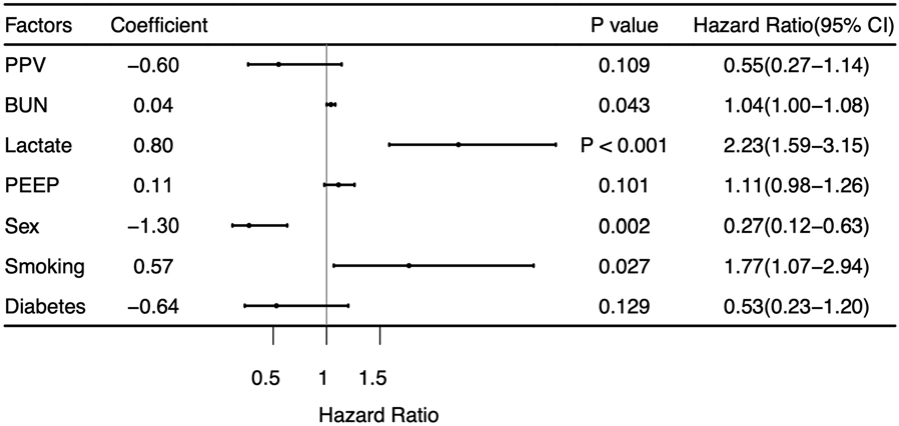
Effect of demographic and disease features on mortality. *PPV* prone positioning ventilation, *BUN* blood urea nitrogen, *PEEP* positive end-expiratory pressure

Figure 5 shows the results of the Cox proportional hazards regression for factors associated with ICU mortality at 28 days. High levels of blood urea nitrogen (HR, 1.04; 95% CI 1.00–1.08; *P* = 0.043) and lactate (HR, 2.23; 95% CI 1.59–3.15; *P* < 0.001) and smoking (HR, 1.77; 95% CI 1.07–2.94; *P* = 0.027) were associated with higher 28-day mortality, and female sex (HR, 0.27; 95% CI 0.12–0.63; *P* = 0.002) was associated with lower 28-day mortality. PPV was not associated with the risk of ICU 28-day mortality (HR, 0.55; 95% CI 0.27 to 1.14,* P* = 0.109). Univariate analysis results of Cox proportional hazards regression was appended as supplementary file 1.

## Discussion

In this observational, retrospective, cohort study, we found that PPV did not improve oxygenation or reduce the mortality of patients with PCP and moderate–severe ARDS. Furthermore, PPV was associated with more invasive mechanical ventilation days and fewer ventilator-free days at 28 days.

To our knowledge, this is the first study examining the effect of PPV on patients with PCP. Our findings are consistent with previous studies showing that patients with ARDS, a heterogeneous syndrome, may show distinct physiological and clinical responses to the same treatment [19–21]. Lung morphology is a promising approach by which ventilation protocols for patients with ARDS can be formulated, and PPV may not be appropriate for every patient as therapies, such as high PEEP and recruitment maneuvers.

Our findings regarding oxygenation evolution during PPV can be explained as follows. First, cases and trials have consistently reported that patients with PCP show GGOs, increased interstitial markings in early stages and patchy consolidation in mid or late stages [7–10], demonstrating a more even distribution of ventilation along the anteroposterior axis than patients with non-PCP ARDS. Previous studies have demonstrated that lung perfusion distribution is similar in the prone and supine positions [22, 23]. Consequently, ventilation of the dorsal lung may change minimally during PPV, eventually resulting in an approximate ventilation/perfusion ratio in the supine position. This finding agrees with a previous study showing that higher PEEP and recruitment maneuvers might be more suitable for patients with nonfocal ARDS, whereas patients with focal ARDS might benefit more from PPV and lower PEEP [24–27].

Second, 58 (73%) patients in our study had coexisting microbes in the lung, leading to nonuniform changes in ventilation in the dorsal lung and ventilation/perfusion ratio that varied across patients resulting in different responses to prone positioning. Third, the LIVE study showed that ventilated patients with nonfocal ARDS in the personalized group that had a high PEEP reached a Pplat of 30 cm H_2_O and recruitment maneuvers [26]. However, in our study, patients were ventilated according to the ALVEOLI study, and recruitment maneuvers were not performed routinely [17, 28, 29], and the collapsed alveolar group may not have been fully recruited compared to nonfocal ARDS in the personalized group of the LIVE study. Thus, in the first PPV session, which was conducted in the first 24 h after ICU admission, ventilation may redistribute and result in considerable decreases in SpO_2_.

Furthermore, most patients in our study received NIV before ICU admission but failed, and many patients showed consolidation and were in the late stage of PCP [3, 10]. The mortality remained high despite standard pharmacotherapy, lung-protective ventilation and even with ECMO.

Our study has several limitations. First, the retrospective nature of the study, together with the absence of Pplat measures at each time phase, may have affected the outcomes observed in this study. Second, lung perfusion and ventilation of PCP may not have been assessed accurately, and ventilation/perfusion matching requires further monitoring in the supine position and prone position. Future studies with a prospective, randomized controlled trial design and with larger sample sizes are therefore needed.

In summary, we found that prone positioning did not improve oxygenation or the survival rate of intubated patients with PCP and moderate–severe ARDS. PPV results in more invasive mechanical ventilation days and is associated with a high incidence of adverse events. Our findings do not support the routine use of PPV in these patients (Additional file 1).

### Supplementary Information

**Additional file 1. **Univariate analysis results of Cox proportional hazards regression.

## Data Availability

The datasets used for the current study are available from the corresponding author upon reasonable request.
