# Supplementary material for: Prone positioning does not improve outcomes of intubated patients with pneumocystis pneumonia and moderate–severe acute respiratory distress syndrome: a single-center, retrospective, observational, cohort study
Source: Eur J Med Res. 2024 May 3;29:267. doi: 10.1186/s40001-024-01868-7 (PMC11067229; doi:10.1186/s40001-024-01868-7)
Supplement: Supplementary file 1 — Additional file 1. Univariate analysis results of Cox proportional hazards regression. [file 40001_2024_1868_MOESM1_ESM.docx]

**Additional file1: univariate analysis results of Cox proportional hazards regression**

| **Variable** | **Coefficient** | **P value** | **HR（95%CI）** |
| --- | --- | --- | --- |
| age | 0.01 | 0.500 | 0.99（0.96, 1.02） |
| BMI | 0.02 | 0.742 | 1.02（0.89, 1.17） |
| PPV | -1.22 | 0.035 | 0.30（0.10, 0.92） |
| CD4 count | -0.004 | 0.420 | 1.00（0.99, 1.01） |
| Albumin | 0.009 | 0.160 | 1.01（1.00, 1.02） |
| LDH | < 0.001 | 0.840 | 1.00（1.00, 1.00） |
| WBC | 0.05 | 0.243 | 1.05（0.97, 1.14） |
| PCT | 0.02 | 0.375 | 1.01（0.98, 1.05） |
| CRP | 0.002 | 0.346 | 1.00（1.00, 1.01） |
| GRAN% | -0.02 | 0.291 | 0.98（0.96, 1.01） |
| BUN | 0.08 | 0.019 | 1.09（1.01, 1.17） |
| Bicarbonate | -0.02 | 0.624 | 0.98（0.92, 1.05） |
| Lactate | 1.11 | < 0.001 | 3.05（1.87, 4.95） |
| PEEP | 0.16 | 0.047 | 1.18（1.00, 1.38） |
| FiO_2_ | 2.16 | 0.228 | 8.63（0.26, 287.62） |
| PaO_2_/FiO_2_ | 0.007 | 0.449 | 1.01（0.99, 1.03） |
| Sex | -1.75 | 0.002 | 0.17（0.06, 0.53） |
| Sepsis | -0.10 | 0.791 | 0.91（0.44, 1.88） |
| Pulmonary consolidation | 0.10 | 0.803 | 1.10（0.51, 2.39） |
| GGO | -0.46 | 0.235 | 0.63（0.30, 1.35） |
| Interstitial marking | 0.33 | 0.421 | 1.40（0.62, 3.15） |
| Smoking | 0.76 | 0.031 | 2.13（1.07, 4.23） |
| Chronic pulmonary disease | -0.80 | 0.121 | 0.450（0.16, 1.24） |
| Chronic kidney disease | -1.03 | 0.098 | 0.36（0.10, 1.21） |
| Diabetes | -0.91 | 0.087 | 0.40（0.14, 1.14） |
| Anti-PCP before ICU | -0.56 | 0.144 | 0.57（0.27, 1.21） |
| Anti-PCP in ICU | -1.93 | 0.129 | 0.15（0.01, 1.76） |

Concordance= 0.759 (se = 0.039)

Likelihood ratio test= 48.41 on 27 df, p=0.007

Wald test = 41.38 on 27 df, p=0.04

Score (logrank) test = 48.93 on 27 df, p=0.006

Factors marked as blue denote those that entered the final multivariate analysis of Cox proportional hazards regression.
